# A cross-sectional study of clinical features of bacterial meningitis among neonates presumed to have sepsis in a tertiary hospital, Dar es Salaam, Tanzania

**DOI:** 10.11604/pamj.2023.46.123.32787

**Published:** 2023-12-29

**Authors:** Taher Pishori, Francis Fredrick Furia, Karim Manji

**Affiliations:** 1 Department of Pediatrics, TMJ Hospital, Dar es Salaam, Tanzania,; 2 Department of Pediatrics and Child Health, School of Medicine, Muhimbili University of Health and Allied Sciences, Dar es Salaam, Tanzania

**Keywords:** Neonatal sepsis, neonatal meningitis, sepsis

## Abstract

Identifying meningitis among neonates is usually challenging given the non-specific presentation and overlap with neonatal sepsis. This study was aimed at determining the pertinent clinical features that would suggest bacterial meningitis among infants with signs of possible serious bacterial infection and their outcomes. This hospital-based cross-sectional study was conducted among neonates presenting with clinical features of sepsis admitted at Muhimbili National Hospital (MNH) between May and December 2015. Detailed clinical features, blood cultures, and cerebrospinal fluid were obtained. The specimens were tested at the Central Pathology Laboratory at MNH. Short-term clinical outcome was also determined for recruited participants. One hundred and twenty-six neonates met the inclusion criteria and were recruited, males were 67 (53.2%) and the mean age of participants was 10.4 ± 7.9 days. Features of meningitis were noted among 19% (24/126) and very low birth weight neonates were observed to have a statistically higher prevalence of meningitis (p=0.038). Bacterial isolates from cerebrospinal fluid (CSF) culture were Klebsiella spp and E. coli, while predominant isolates from blood culture were Klebsiella spp (35%) and E. coli (20.6%). There was high resistance to ampicillin (91.2%), cloxacillin (94.1%), gentamycin (50%), and ceftriaxone (50%). A high mortality of 24.9% was noted. Neonatal meningitis is common among neonates with sepsis, and bacterial isolates were resistant to routinely used antibiotics. High mortality attributed to meningitis was noted at Muhimbili National Hospital.

## Introduction

Meningitis is a form of sepsis that contributes to high morbidity and mortality among neonates globally and the burden of this condition is high in low-income countries including Tanzania [1,2]. Most affected neonates include those with low birth weight, prematurity, and maternal colonization with group B streptococcus (GBS), the most common offending organisms include GBS, *E. coli, Klebsiella spp* listeria monocytogenes [3-5].

Neonatal sepsis is attributed to many factors including maternal colonization, and because of low immature immune systems, these babies are at increased risk for meningitis [6,7]. Characterizing the clinical profile of meningitis among neonates is valuable in managing affected babies, the existing lacunae in the information of this condition was the reason for conducting this study at Muhimbili National Hospital.

## Methods

**Study design, setting, and participants:** this cross-sectional study was conducted in the neonatal ward at Muhimbili National Hospital from May to December 2015. All neonates admitted in the neonatal ward meeting the World Health Organization (WHO) definition of neonatal sepsis [8] were eligible, 126 met the criteria for neonatal sepsis and were recruited consecutively.

**Sample size:** Kish and Leslie formula using a prevalence of 17.9% neonatal meningitis reported by Laving *et al*. from a study conducted in Kenya was used for sample size calculation, a margin error of 5% and a 95% confidence interval was utilized [5].

**Variables:** the main variables for this study were clinical meningitis, aetiological pathogens, and antimicrobial susceptibility patterns of isolates. Meningitis was defined by either cell count abnormality in the CSF (white cell count > 32/mm^3^ for term and > 29/mm^3^ for preterm) or positive biochemical results of CSF specimen (proteins > 1.7g/dl for preterm babies or > 1.5g/dl for term babies, CSF glucose < 50% of random blood glucose test (RBG test) or isolation of bacterial pathogens from culture of CSF specimens.

**Measurements:** structured questionnaires were used to collect data including demographic, clinical presentation, and outcome. Weight was measured using SECA beam balance to the nearest 100 g. From each participant, 2 ml of venous blood was aseptically drawn from the anterior cubital fossa of each neonate and inoculated into pediatric blood culture bottles. A lumbar puncture was performed aseptically to obtain cerebral spinal fluid (CSF) and concurrent random blood glucose was also checked using the ACCU-CHEK Active machine.

Cerebral spinal fluid (CSF) glucose and protein were determined by the use of an ARCHITECT c4000 clinical chemistry analyzer. Cell count was carried out by a counting chamber that is an improved Neubauer Chamber. CSF specimens were inoculated onto solid agar plates; MacConkey, blood, and chocolate agars for up to 48 hours before being regarded as no growth. Blood culture bottles were incubated at 37°C for 24 hours after which aliquots were sub-cultured on solid agar plates; MacConkey, blood, and chocolate agars for up to 96 hours before being regarded as having no growth. Colonies on solid agar plates were identified based on characteristic morphology, gram stain appearance, and standard commercially prepared biochemical tests.

Antimicrobial sensitivity testing was performed for routinely used antimicrobials for treating neonatal sepsis at MNH, drug concentrations used were; ampicillin 10µg, cloxacillin 30µg, gentamicin 10µg, ceftriaxone 30µg, amikacin 30µg and vancomycin 30µg, results were recorded as resistance and sensitive [7]. Bacterial meningitis was defined by either cell count abnormality in the CSF (white cell count > 32/mm^3^ for term and > 29/mm^3^ for preterm) or positive biochemical results of CSF specimen (proteins > 1.7g/dl for preterm babies or > 1.5g/dl for term babies, CSF glucose < 50% of RBG) or isolation of bacterial pathogens from culture of CSF specimens.

**Statistical methods:** Package for Social Sciences (SPSS) was used for data entry, cleaning, and analysis using the Chi-square test and Fishers' exact test, a p-value of < 0.05 was considered statistically significant.

**Ethical considerations:** Muhimbili University of Health and Allied Sciences (MUHAS) ethical review board and MNH administration provided approval and permission respectively for this study. Informed consent was obtained from the parents/guardians before recruitment.

## Results

**Maternal and neonatal demographic and baseline characteristics of the study population:** one hundred and ninety-five neonates were screened, 69 were excluded; 32 refused consent, 17 had neural tube defects, 20 had a septic shock with disseminated intravascular coagulopathy and lumbar puncture could not be performed. Out of 126 neonates who were included in the study, 67 (53.2%) were male and the mean age was 10.4 ± 7.9 days ranging from 2 to 24 days. Sixty-five (51.5%) neonates had low birth weight, and suspected sepsis were admission reasons for 35 (27.8%) and 75 (60%) neonates respectively. The majority of neonates (64.3%) were delivered by Spontaneous Vaginal Delivery (SVD) and foul-smelling amniotic fluid was noted in 17/126 (13.5%) deliveries. Five out of 126 (4%) mothers tested positive for HIV during pregnancy.

**Clinical features of study participants:** tachypnea (72.2%), lethargy/coma (66.7%), difficulty in feeding (65.1%), fever (63.5%), severe chest in-drawing (58.7%) convulsion (54.8%), and skin mottling/sclerema (51.6%). Other clinical features included hypothermia (36.1%) and bulging fontanel (4.8%).

**The proportion of children with features of meningitis among the septic infants (Table 1):** twenty-four participants (19%) had features of meningitis, of which 3 (12.5%) had bacterial isolation from CSF, 11 (45.8%) had aseptic CSF, raised protein, and low glucose levels in CSF while 10 (41.7%) leukocytosis on CSF microscopy. Neonates with very low birth weight were noted to have a higher prevalence of meningitis (37.5%) as compared to those with low birth weight (14.6%) and normal birth weight (14.8%), p = 0.038 (Table 2). The mean CSF protein and mean cell count were significantly higher in children with meningitis as compared to those without (p < 0.001), while the mean CSF glucose level was lower in those with features of meningitis (p < 0.001).

**Table 1:** baseline characteristics and factors associated with neonatal meningitis

Variable	Meningitis	No-meningitis	Total	p-value
	n (%)	n (%)	n (%)	
**Gestation age (weeks)**				
Less than 37	11 (21.2)	41 (78.8)	52 (41.3)	
37 to 42	13 (17.5)	61 (82.4)	74 (58.7)	0.614
**HIV status**				
Positive	0 (0)	5 (100)	5 (4)	
Negative	24 (19.8)	97 (80.2)	121 (96)	*0.582
**Mode of delivery**				
Spontaneous vertex delivery	14 (17.3)	67 (82.7)	81 (64.3)	
Emergency caesarean section	10 (23.8)	32 (76.2)	42 (33.3)	0.685
Elective caesarean section	0 (0)	1 (100)	1 (0.8)	
Assisted birth delivery	0 (0)	2 (100)	2 (1.6)	
**Duration of labour (hours)**				
Less than 12	4 (13.3)	26 (86.7)	30 (36.1)	0.518
More than 12	10 (18.9)	43 (81.1)	53 (63.9)	
**Liquor**				
Clear	16 (18.4)	71 (81.6)	87 (69)	
Meconium stained	6 (27.3)	16 (72.7)	22 (17.5)	0.455
Foul-smelling	2 (11.8)	15 (88.2)	17 (13.5)	
**Age of neonate (week)**				
Less than a week	11 (17.5)	52 (82.5)	63 (50)	0.650
Two and more	13 (20.6)	50 (79.4)	63 (50)	
**Sex**				
Male	13 (19.4)	60 (80.6)	67 (53.2)	0.914
Female	11 (18.6)	42 (81.4)	59 (46.8)	
**Reasons for admission**				
Premature	3 (8.6)	32 (91.4)	35 (27.8)	
Birth asphyxia	0 (0)	9 (100)	9 (7.1)	
Congenital malformation	1 (14.3)	6 (85.7)	7 (5.6)	0.102
Sepsis	18 (28.6)	45 (71.4)	63 (50)	
Hypothermia	1 (11.1)	8 (88.9)	9 (7.1)	
Fever	1 (33.3)	2 (66.7)	3 (2.4)	
**Birth weight**				
Very low birth weight	9 (37.5)	15 (62.5)	24 (19)	
Low birth weight	6 (14.6)	35 (85.4)	41 (32.5)	0.038
Normal birth weight	9 (14.8)	52 (85.2)	61 (48.4)	
**Duration of hospitalization (weeks)**				
1-2 weeks	3 (20)	42 (56.8)	45 (50.6)	
More than 2 weeks	18 (80)	32 (43.2)	44 (49.4)	0.009
**Seizures at discharge**				
Present	3 (12.5)	14 (13.7)	17 (13.5)	
Absent	21 (87.5)	88 (86.3)	109 (86.5)	0.923
**Death**				
Discharged alive	15 (62.5)	74 (72.5)	89 (70.6)	
Died in hospital	9 (37.5)	28 (27.5)	37 (29.4)	0.331

* Fisher exact test

**Table 2:** sensitivity pattern of bacterial isolates from blood

Bacteria isolated	VANC	CEFT	GENT	CLOX	AMP	AMIK
	*S (T)	*S (T)	*S (T)	*S (T)	*S (T)	*S (T)
*E. coli*	3 (7)	4 (7)	5 (7)	0 (7)	0 (7)	6 (7)
*Pseudomonas Spp*	0 (3)	0 (3)	0 (3)	0 (3)	0 (3)	3 (3)
*S. Aureus*	6 (6)	2 (6)	2 (6)	0 (6)	1 (6)	3 (6)
*Klebsiella Sp*	2 (12)	8 (12)	8 (12)	0 (12)	0 (12)	11 (12)
*CoNS*	4 (4)	2 (4)	2 (4)	1 (4)	1 (4)	2 (4)
*S. Pneumoniae*	1 (2)	1 (2)	1 (2)	1 (2)	1 (2)	1 (2)

* S (T): sensitivity to drug (total bacteria isolated)

**Isolated organisms from blood and CSF culture (Table 2):** bacteria were isolated from 34 blood culture and three CSF specimens, blood isolates included *Klebsiella spp* (35.3%), *E. coli* (20.6%), *S. aureus* (17.6%) coagulase-negative Staphylococcus (11.8%), *Pseudomonas spp* (8.8%), and *S. pneumoniae* (5.9%) while CSF isolates included *E. coli* (2 specimens) and *Klebsiella spp*. Two neonates had similar organisms isolated from blood, and CSF growing *E. coli* and *Klebsiella spp*. One neonate had isolation of *E. coli* from CSF and *S. aureus* from blood.

Sixteen neonates out of 34 (47%) with positive blood culture had features of coagulase-negative staphylococci (CNS) involvement while 58.3% (14/24) of those with features of meningitis had positive growth on blood culture. Only 3 (8.8%) and 2 (5.9%) out of all blood isolates were sensitive to ampicillin and cloxacillin respectively, and 18/34 (53%) isolates were sensitive to gentamicin. The three isolates from CSF were resistant to ampicillin, cloxacillin, ceftriaxone, gentamycin, and vancomycin, they were only sensitive to amikacin.

**Outcome of study participants:** the overall mortality rate was 29.4%, it was higher among neonates with features of meningitis as compared to those without (37.5% vs 27.5%) although there was no statistically significant difference (Table 1). Seizures were noted in 12.5% (3/24) and 13.7% (14/102) of neonates with and without features of meningitis respectively. Hospital stay was longer for neonates with features of meningitis, with 80% staying for more than 2 weeks as compared to 43% of those without (p- value 0.009).

## Discussion

There is a significant burden of meningitis among neonates in our setting, with common aetiology comprising of *E. coli* and *Klebsiella spp*, prolonged hospital stay and higher mortality was noted among neonates with meningitis in this study, similar to other reports in the region [4,5]. Commonly reported risk factors for neonatal meningitis including premature rupture of membranes and stained liquor did not show any influence on meningitis in this study, highlighting the possibility of these infections even in the absence of known risk factors [5]. Low birth weight and premature who have low immunity were noted to be more vulnerable to meningitis in this study, which is agreement with existing literature [9,10].

Positive CSF culture was noted in only three out of 24 neonates with features of meningitis, majority of which (70%) were delivered in other facilities, and had antibiotics before referral to MNH. Delayed lumbar puncture and use of antibiotics may have contributed to poor yield, making it important to improve capacity for evaluating neonates in primary care level in neonates with presumed sepsis, and to limit the use of treatment based on clinical judgment [11].

Concordant bacterial isolates from CSF and blood were noted for *E. coli* and *Klebsiella spp* in this study, these are commonly reported causative organisms for neonatal meningitis [3-5]. This growth emphasized considering meningitis for neonates with sepsis. Other organisms isolated from blood culture were similar to findings from other studies conducted in Tanzania [6].

High resistance to routinely used antibiotics and those recommended by WHO for treatment of neonatal sepsis was noted, this is similar to other reports in the region [6]. High mortality and prolonged hospital noted in this study, particularly among neonates with features of neonatal meningitis, and high resistance of isolates call for robust community-based studies to examine applicability of the current antibiotic recommendations for neonatal sepsis.

**Limitations:** this was a single-center hospital-based study therefore findings may not be generalizable to the community setting.

**Funding:** this study was funded by Fogarty Global Infectious Disease (GID) training grant (NIH/Fogarty GID grant number: 2 D43 TW007886-05).

## Conclusion

Features of meningitis were common among neonates with presumed sepsis, bacterial isolates from CSF included *E. coli* and *Klebsiella spp* while *Klebsiella spp, E. coli*, and *S. aureus* were isolated from blood. High resistance to ampicillin, cloxacillin, and gentamycin were noted. Participants with features of meningitis had prolonged hospital stays and high mortality.

### What is known about this topic

- *Neonatal infections are common and contribute to mortality*.

### What this study adds

- *Central nervous system involvement is common with neonatal sepsis;*
- *There is high mortality with central nervous system involvement;*
- *There is high resistance to organisms isolated from cerebral spinal fluid*.
